# Antiproliferative activity of standardized herbal phytopreparation from *Asclepias subulata*

**DOI:** 10.12688/f1000research.111181.1

**Published:** 2022-05-16

**Authors:** Francisco Humberto González Gutiérrez, Luisa Alondra Rascón Valenzuela, Salvador Enrique Meneses Sagrero, Marcelo J. Dias-Silva, Olivia Valenzuela Antelo, Carlos Velazquez, Wagner Vilegas, Ramón Enrique Robles Zepeda

**Affiliations:** 1 Ciencias Químico Biológicas, Universidad de Sonora, Hermosillo, Sonora, 83000, Mexico; 2 Universidade Estadual Paulista (UNESP), Sao Vicente, Sao Paulo, Brazil

**Keywords:** Asclepias subulate, Calotropin, Cardenolides, Standardized extract, Antiproliferative activity

## Abstract

**Background:** Several studies have shown that active compounds of *Asclepias subulata* (cardenolides) have antiproliferative effect on human cancer cells. Cardenolides isolated from *A. subulata* can be used as active chemical markers to elaborate phytopharmaceutical preparations. To evaluate the antiproliferative effect of a standardized extract of the aerial parts, based on *Asclepias subulata* cardenolides.

**Methods: **Four standardized extracts were prepared by HPLC-DAD depending on the concentration of calotropin and the antiproliferative activity was measured for the MTT assay, on the A549, MCF-7, HeLa, PC3 and ARPE cell lines. The concentrations of calotropin used for the standardization of the extracts were 10, 7.6, 5 and 1 mg/dL.

**Results:** Standardization of the *A. subulata* extract based on calotropin at 7.6 mg/g dry weight was achieved and the antiproliferative activity was evaluated over A549, HeLa and MCF-7 cell lines, obtaining proliferation percentages of 3.8 to 13.4% *.*

**Conclusions:** The standardized extracts of *A. subulata* at different concentrations of calotropin showed antiproliferative activity against all the cell lines evaluated. The greatest effect was observed against the HeLa cell line.

## Introduction

Cancer continues to be the most aggressive disease and with a high mortality rate. World Health Organization (WHO) estimated that during 2015, 8.2 million people died due to this condition ^1^ but the burden increased in 2018 to 18.1 million new cases and 9.6 million deaths. ^2^ In recent years, there is an increasing interest in the study of natural resources for medicine, so research on the phytochemical, pharmacological and clinical validation of numerous active ingredients derived from natural products has been multiplied. ^3^ Natural products have been considered as a significant source for the generation of pharmaceutical compounds such as analgesics, antifungals, antibiotics, and anticancer agents. ^3^ Herbal medicine has become a non-toxic, safe and readily available source of compounds for cancer treatments ^2^ and the design and research of standardized extracts (preparations obtained only from medicinal herbs that contains pharmacologically active components) can generate advances in the study of new therapies against cancer, reducing or avoiding the appearance of side effects ^3^ since it has been proven that herbal medicine are a safe source, not toxic and readily available cancer treatment compounds. ^2^

*Asclepias subulata* (Asclepiadaceae) is a native plant from the Sonoran Desert in North America, which has been used in traditional medicine for the treatment of several diseases and cancer types. ^4^ In our research group *Rascon et al.,* 2015, ^5^ were able to demonstrate the antiproliferative activity of the ethanolic extract of *A. subulata* the aerial parts of, obtaining results of IC _50_ of 0.4 and 8.4 μg/mL in A549 and HeLa respectively. In addition, they demonstrated the apoptotic activity of the plant extract. These authors also fractionated the extract to obtain the bioactive components, obtaining a new cardenolide 12,16-dihydroxycalotropin and three known compounds, calotropin, corotoxigenin 3-O-glucopyranoside and desglucuzarina. All these compounds showed high antiproliferative activity and selectivity in human cancer cells. ^4^ Despite the most important use of the cardiac glycosides are on cardiac failures treatments, ^6^^,^^7^ recent studies evidenced that cardenolides are also apoptosis inductors and growth-inhibitors against tumors in *in vitro* and *in vivo* models, with no significant toxicity on normal cells. ^6^^,^^8^ These data stimulated us to generate a standardized extract of the aerial parts of *A. subulata* based on its most abundant cardenolides of the plant.

## Methods

### Plants material

*A. subulata* (Decne., 1844) (Asclepiadaceae) was collected in an experimental area located in the Department of Agriculture and Livestock (DAL) of the University of Sonora, Mexico (29°03′18″ North latitude and 111°05′21″ West longitude). The taxonomic identification was carried out by Eng. Jesus Sanchez Escalante and deposited in the Herbarium of the University of Sonora with the voucher number USON-26395.

### Obtaining ethanolic extracts (AsE)

The aerial parts of *A. subulata* was dried at room temperature (25°C), in the shade and grounded with a Whiley mill (200 mesh) affording 300 g of the powder, that was macerated with 70% ethanol in a 1:10 w/v proportion with manual agitation for 10 days. The ethanolic extract was filtered and concentrated on a rotary evaporator at 40 °C under reduced pressure affording 172 g (57.4% rendimento) of the *A. subulata* etanolic extract (AsE).

### Design and preparation of standardized extracts

The cardenolides were identified and isolated following the methodology described by Rascon et al., 2015. ^4^ The standardization was performed using a High Performance Liquid Cromatograph (HPLC) (Jasco system compound of binary pump model PU-2086 Plus, São Paulo, SP, Brazil) coupled to a diode array detector (DAD) (Jasco MD-2018 Plus, São Paulo, SP, Brazil) and to an evaporative light scattering detector (ELSD) (Jasco ELS-2040, São Paulo, SP, Brazil). We use a Luna C18 (2) 100A column (250 × 21.2 mm d.i, 5 μm) (Phenomenex, CA, USA) with pre-column (4 × 3 mm d.i.) were used. A binary solvent system was used; solvent A (water with 0.1% formic acid); and solvent B (methanol with 0.1% formic acid) at a flow rate of 7 mL/min ^4^. The run was monitored at 30 min. The identification of the chromatographic peaks was performed using addition of compound isolated in the previous step. ESI-IT-MS/MS spectra were obtained with a LTQXL Thermo Scientific spectrometer (ionization mode: negative or positive, scan range: 150–2000 m/z, voltage: –13 kV, heated capillary temperature: 280°C, sheathgas:10 μa, auxiliarygas:10 μa) (San Jose, CA, USA). The NMR experiments were performed on a Bruker Advance 600 MHz equipment, and the samples were processed using CD3OD as the solvent using one and 2D techniques (DEPTQ, HMBC, HSQC, and TOCSY) to confirmation of the structures of the isolated compounds. The calibration curve was prepared using the standard addition method, with seven different concentrations of calotropin (0.3-1 mg/mL) and the values were projected in graph using the PRISMA 5. Considering the IC _50_ of the *in vitro* antiproliferative activity observed in our previous works, ^5^ four standardized extracts were prepared by HPLC-DAD depending on the concentration of calotropin and the antiproliferative. The concentrations of calotropin used for the standardization of the extracts were 10, 7.6, 5 and 1 mg/dL, taken by previous studies. ^4^^,^^5^

### Cells lines

A549 (human alveolar adenocarcinoma) (ATCC number: CCL-185), PC-3 (human prostatic adenocarcinoma) (ATCC number: CRL-1435), LS180 (human colorectal adenocarcinoma) (ATCC number: CL-187), HeLa (human cervix adenocarcinoma) (ATCC: CCL-2), ARPE-19 (human retinal pigmented epithelium) (ATCC number: CRL-2302) cell lines were purchased from the American Type Culture Collection (ATCC, Rockville, MD, USA). The cell lines were maintained using Dulbecco’s Modified Eagle’s Medium culture medium supplemented 5% of fetal bovine serum. Cells were grown incubated at 37 °C in a humidified incubator with 5% of CO _2_.

### Antiproliferative activity assay

Antiproliferative activity was evaluated by the MTT reduction assay [3-(4,5-dimethylthiazol-2-yl)-2,5-diphenyltetrazolium] with some modifications. ^5^ Briefly, the cells were added into a 96-well plate (1 × 10 ^4^ cells per well in 50 μL of medium). After 24 h incubation at 37°C, were added 50 μL of medium containing different concentrations of standardized extracts: 10 mg/dL; 7.6 mg/dL; 5 mg/dL and 1 mg/dL (maximal DMSO concentration were of 2% v/v) and the cell cultures were incubated for 48 h. Doxorubicin was used as positive control. In the last 4 h of incubation time the cells where were washed with PBS 1X. Fresh culture medium (100 mL) and 10 μL of MTT solution (5 mg/mL) were added to each well. The formed formazan crystals were dissolved with 100 mL of acidic isopropyl alcohol. The absorbance of the samples was measured with an ELISA plate reader (iMark Microplate Absorbance Reader, Bio-Rad), The differences in means were analyzed using one-way analysis of variance (one-way ANOVA) followed by Tukey’s test (Sigma Stat 3; Systat Software Inc., CA, USA).

## Results

### Obtaining ethanolic extracts (AsE)

**Figure 1.**
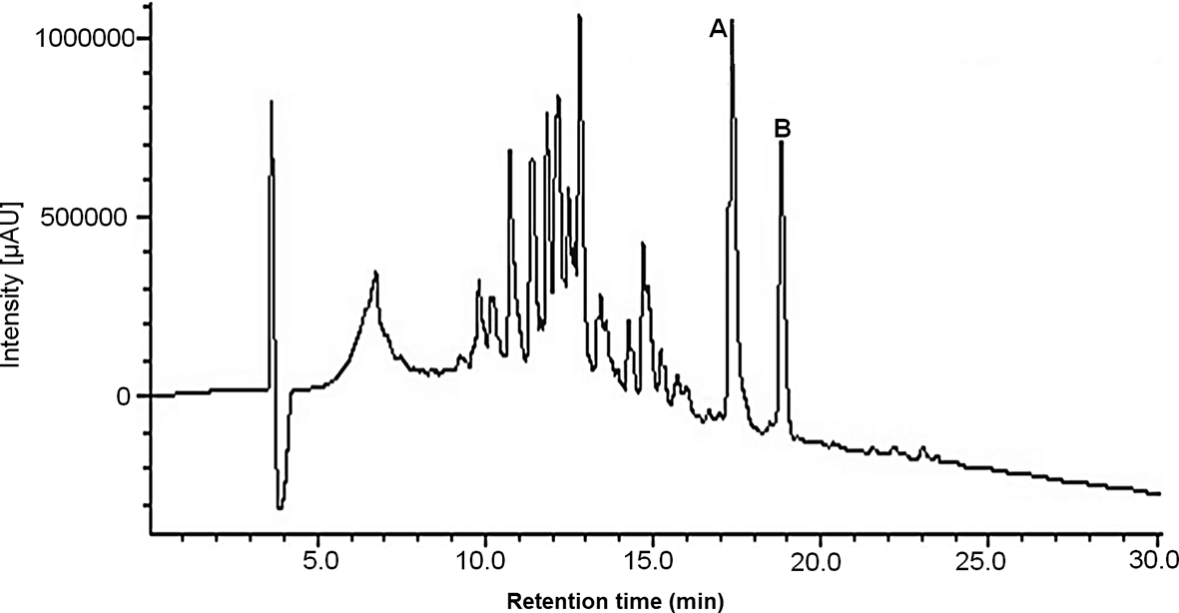
High-Performance Liquid Chromatography chromatogram of *Asclepias subulata* ethanolic extract. A: Calotropin (17.4 min), B: Calactin (18.1 min).

The AsE was prepared by maceration for 10 days with 70% ethanol, filtered and dried, were yielding of 172.1 g (57.4%). Fractionation of 5 g of AsE using gel permeation chromatography on an open column of Sephadex and further purification using a semipreparative HPLC-DAD system led to the isolation of the cardenolides ( Figure 1), which were identified using NMR, Mass spectrometry and comparison to previous literature data. ^4^ Total of 9 subfractions were obtained and then were analized for subjected thin layer chromatography (TLC) technique and HPLC to know the complexity of its compounds. Thus, fractions 3 (1167.2 mg) and 4 (576.6 mg) were selected, which gave us two signals called compound A and compound B, which also demonstrated that their maximum absorption points are between 210 and 225 nm, searching the literature for note that the signals that show a characteristic unique absorption maximum between 214 and 222 nm, corresponding to a functional group of unsaturated lactone and can be considered cardenolides; ^9^ the structures were confirmed by NMR and agree with the data reported by the bibliography. ^4^ The chromatographic profiles were optimized by means of analytical HPLC and subsequently the conditions used were extrapolated to a semi-preparative HPLC system using the ELSD as the main detector due to the low absorption in the UV-visible spectrum that the samples presented. This way it was possible to obtain compounds 1, 2 and 3. Compound 1 (Rt 17.4 min **)** was obtained as a fine white powder (32.8 mg). Its ESI-MS mass spectrum showed an [M-H] ^-^ ion at m/z 531; comparison with the literature allowed us to identify 1 as calotropin. ^4^ Compound 2 (Rt 18.1 min) generated amorphous and colorless crystals with a weight of 21.2 mg, Its ESI-MS mass spectrum showed an [M-H] ^-^ ion at m/z 577; comparison with the literature allowed us to identify 2 as calactin. ^10^ Compound **3 (**R _T_ 17.9 min) was obtained as a fine white powder (5 mg). Its ESI-MS mass spectrum showed the [M-H] ^-^ ion at m/z 547. MS3 fragmentarion of the precursos ion at m/z 547 led to the product iosn at m/z 529, 511 and 401, which are in accordance to Rascon et al; 2015. ^4^ This compound corresponds to cardenolide 4′-hydroxy-7,8-dehydrocalotropin. All these compounds were also subjected to NMR analyses and compared to literature data ^4^ in order to confirm their identities (see extended data for more information ^17^). Continuing with the study, by means of mass spectrophotometry the identification of 11 compounds shown in Supplementary material 2 (see extended data ^17^) was achieved.

### Design and preparation of AsE

**Figure 2.**
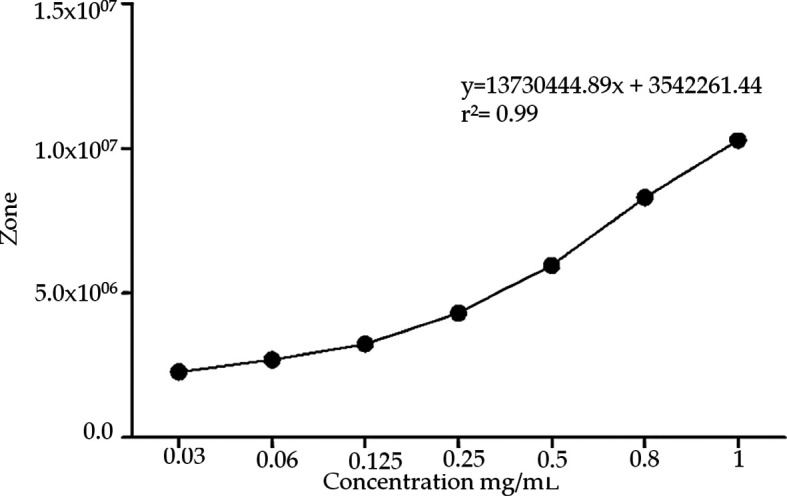
Calibration curve of phytopreparation from *Asclepias subulata.* Calotropin was quantified using different concentrations (0.3-1 mg/mL) whit standard addition method by HPLC.

Plants in a natural environment face different biological and ecological stimuli that mean that they do not provide secondary metabolites in a consistent way; since nature does not provide a product with consistent or standardized concentrations, it is necessary to perform a standardization of extracts that are required to be used in traditional medicine, but the standardization of an herbal product is complicated, starting from its cultivation, extraction, storage, etc. ^11^ After the clean-up, the AsE was analysed using HPLC-DAD-ELSD ( Figure 1). The chromatographic run was achieved in about 30 min, and led to a good resolution of the peaks assined to compounds 1 and 2, which ios essential for the standardization of the AsE. Standardization of the AsE led to the final concentration of 7.6 mg of calotropin per gram of AsE ( Figure 2). Once this information was gathered, we were able to use three different concentrations of the extract based on calotropin and IC _50_ and the IC _50_ reports present in the previous studies: a low (1 mg/dL), a medium (5 mg/dL), a high (10 mg/dL) and a base 7.6 mg/dL that allowed us to continue with the evaluation of the extract in subsequent experiments.

Antiproliferative activity of the AsE

Then, based on this result and on the IC _50_ and the IC _50_ reports reported in our previous studies we were able to investigate the antiproliferative activity of AsE using four different concentrations: 10 mg/dL (high); 7.6 mg/dl (base); 5 mg/dL (medium) and 1 mg/dL (low), which were evaluated in different cell lines ( Table 1), HeLa being the most affected; a maximum of 10% proliferation was observed in all lines, which shows that the extract even in the low concentration of 1 mg/dL stops the growth of cancer cells *in vitro.*

**Table 1.** Percentage of proliferative activity of the phytopreparations of *Asclepias subulata.*

Concentration of calotropin in phytopreparations		Cell Lines (% Proliferation)		
	ARPE-19	A549	HeLa	MCF-7
10 mg/dL	37.0 ± 1.6 ^a^	7.9 ± 0.7 ^b^	4.4 ± 0.6 ^b^	10.2 ± 0.7 ^b^
7.6 mg/dL	38.1 ± 1.1 ^a^	7.9 ± 0.6 ^b^	5.1 ± 0.9 ^b^	10.6 ± 0.8 ^b^
5 mg/dL	42.6 ± 2.1 ^a^	9.0 ± 0.9 ^b^	5.3 ± 1.0 ^b^	11.1 ± 1.0 ^b^
1 mg/dL	47.2 ± 0.9 ^a^	10.0 ± 0.7 ^b^	6.0 ± 0.9 ^b^	11.4 ± 1.0 ^b^

A549. Non-small cell lung cancer (NSCLC). MCF-7. Breast adenocarcinoma. HeLa. Adenocarcinoma of the cervix. ARPE-19: retinal pigment epithelium.

^a^ Statistically significant difference at *p*=0.05. ± Standard deviation.

^b^ There is no statistically significant difference *p*=0.05.

## Discussion

The term cancer includes more than 100 different types of diseases that feature the accelerated and disorderly growth of cells with abnormal expressed genes that participate in the regulation of the cell cycle directly, ^12^ with an incidence of 439.2 cases per 100 thousand inhabitants of which 163.5 will die from the disease. ^1^ The generation of standardized extracts that support the treatments against this disease and given its high mortality and worldwide incidence is of vital importance. In the present work, it was standardized by HPLC-DAD technique, using calotropin as internal standard; the AsE at a concentration of 7.6 mg per dry gram of plant, which presented an antiproliferative activity of less than 1 μg/mL, showing a cytotoxic effect in different cell lines, the most sensitive lines were HeLa and A549, Table 2. The selectivity of the extract for cancer lines is evident when comparing the activity in Table 2, since the ARPE-19 line (non-cancerous line used as control) has a lower sensitivity than cancer lines, this can be tried to explain since the cancer cells, being in a state of massive and uncontrolled proliferation, need different signaling pathways to support their high proliferation metabolism, conveniently changing their energy production from one pathway to another, such as the overexpression of Na ^+^/K ^+^ pumps; epidermal growth receptors (EGFR), insulin receptors, etc. ^13^ Therefore, it is hypothesized that the components present in the *A. subulata* extract could be inhibiting some growth receptors over-expressed in these cell lines, such as EGFR. Rascon *et al*, 2015 reported the antiproliferative activity by IC _50_ of a methanol extract in A549 (8.7 μg/mL) and HeLa (<0.4 μg/mL) cell lines; ^5^ in the present study, an increase in activity was achieved following the activity patterns reported by Rascon *et al*. 2015 reporting the increase in antiproliferative activity when using an ethanolic fraction, ^5^ which was verified by generating an ethanolic extract and observing the increase in antiproliferative activity at 0.23 μg/mL in A549 and 0.6 in HeLa, respectively, Table 1. This is due to the concentration of secondary metabolites (cardenolides) in this fraction. The comparison between the antiproliferative activity of a wild *A. subulata* extract and an extract from artificial culture is demonstrated by comparing the works of Bustamante *et al.,* 2020 (IC _50_ 0.8 μg/mL), ^14^ Rascon *et al.,* 2015 (IC _50_ 8.7 μg/mL) ^5^ and the present work (IC _50_ 0.23 μg/mL); the wild plant, being in conditions of natural stress, does not modify the production of its secondary metabolites, but in a crop where these stress levels are varied, it can choose the best growing conditions for the plant was harvested and thus generate a better extract with better antiproliferative activity. ^14^^,^^15^ In the study by Bustamante *et al.,* 2020 showed a calotropin production (reference cardenolide) of 236. 97 μg/g in average of the generated crop, ^14^ while in this study a concentration of 7.6 mg of calotropin per dry gram of plant is reported.

**Table 2.** Antiproliferative activity of the ethanolic extract of *Asclepias subulata.*

	ARPE-19	Cell Lines (IC _50_ μg/mL)		
		A549	HeLa	MCF-7
Ethanolic extract of *Asclepias subulata*	>20.0	0.23 ± 0.03	0.6 ± 0.2	0.5 ± 0.03
Doxorubicin *	1.02 ± 0.12	1.7 ± 0.12	>4.0	0.7 ± 0.04

A549. Non-small cell lung cancer (NSCLC). MCF-7. Breast adenocarcinoma. HeLa. Adenocarcinoma of the cervix. ARPE-19: retinal pigment epithelium. ± Standard deviation.

* Positive control.

This high difference is being influenced by different biotic stress factors such as the herbalism of the plant by worms of the monarch butterfly and the false monarch at the time of the collection of the plant; Agrawal *et al.* 2014 established that the increase in the production of cardenolides in *Asclepias sicaria* and *Asclepias halli* when they are stimulated by herbalism of monarch butterfly worms, thus showing that the plant increases the production of cardenolides as a defense mechanism. ^15^ The method of quantification of cardenolides is another factor to consider, for his part Bustamante *et al.,* 2020 uses an external standardization pattern while in the present work an internal one was used, managing to eradicate the error of the matrix. It has been shown that the antiproliferative activity of the extracts from *Asclepias subulata* given by its bioactive principles the cardenolides such as: calotropin, calactin; 4′-hydroxy-7,8-dehydrocalotropin; identified in the plant (supplementary material 2) ^4^ that can interact with the Na ^+^/K ^+^ pump and cell death receptors, caspase activation and mitochondrial membrane depolarization, among other signaling pathways for the generation of cellular apoptosis. ^8^^,^^16^ Studies by Rascon *et al.* 2015 demonstrated that the *Asclepias subulata* extract could induce cell apoptosis through the activation of caspases 3, 8 and 9 and the depolarization of mitochondria, hypothesizing that the activation of apoptosis occurs intrinsically. ^4^^,^^5^^,^^8^ More recent studies have shown that cardenolides can activate various metabolic pathways that induce apoptosis, the most defined mechanism being the interaction they have with the sodium potassium pump, since when interacting with it they generate a conformational change that induces the activation of the protein. c-Src tyrosine kinase, which interacts with the epidermal growth receptor (EGFR) and the activation of the Ras-Raf pathway by activating the protein by phosphorylation Ras which in turn initiates the downstream signaling pathway of Raf, Mek and Erk that leads to the production of reactive oxygen species and activation of pro-apoptotic proteins such as Bax and the decline of anti-apoptotic proteins of the BCL-2 family, the depolarization of the mitochondrial membrane and activation of caspases, activation of cycle arrest proteins in cells such as p53 and p21 that together act for the activation of cellular apoptosis. ^4^^,^^5^^,^^8^^,^^9^ It is hypothesized that this pathway is the mechanism of action of the generated extract and to achieve its clarification it is of vital importance to evaluate the proteins involved such as p53, Bax, Ras, Raf among others to establish it.

## Conclusions

An ethanolic extract of *Asclepias subulata* was generated with a concentration of 7.6 mg of calotropin per gram of dry weight with IC _50_ less than 1 μg/mL in different cell lines, presenting better activity in cell lines such as HeLa and A549, which places it as an extract with activity antiproliferative candidate for generation of phytopreparation against cancer.

## Data availability

### Underlying data

Open Science Framework: Antiproliferative activity of standardized herbal phytopreparation from Asclepias subulate, https://doi.org/10.17605/OSF.IO/NC5V3. ^17^

This project contains the following underlying data:

- Mass data.rar
- Francisco_4′-hydroxy-7,8-dehydrocalotropin.zip
- Francisco_Calactin.zip
- Francisco_Calotropin.zip
- Calibration curve data

### Extended data

Open Science Framework: Antiproliferative activity of standardized herbal phytopreparation from Asclepias subulate, https://doi.org/10.17605/OSF.IO/NC5V3. ^17^

This project contains the following extended data:

- Compounds identified by HPLC-MS from ethanolic extract of Asclepias subulata.docx
- Spectrometric data from 1H-RMN y 13C-RMN for compounds A and B in CD3OH δ expressed in ppm.docx

Data are available under the terms of the Creative Commons Attribution 4.0 International license (CC-BY 4.0).
