# Antiproliferative activity of standardized herbal phytopreparation from *Asclepias subulata*

**DOI:** 10.12688/f1000research.111181.2

**Published:** 2022-10-25

**Authors:** Francisco Humberto González Gutiérrez, Luisa Alondra Rascón Valenzuela, Salvador Enrique Meneses Sagrero, Marcelo J. Dias-Silva, Olivia Valenzuela Antelo, Carlos Velazquez, Wagner Vilegas, Ramón Enrique Robles Zepeda

**Affiliations:** 1 Ciencias Químico Biológicas, Universidad de Sonora, Hermosillo, Sonora, 83000, Mexico; 2 Universidade Estadual Paulista (UNESP), Sao Vicente, Sao Paulo, Brazil

**Keywords:** Asclepias subulate, Calotropin, Cardenolides, Standardized extract, Antiproliferative activity

## Abstract

**Background: **Several studies have shown that active compounds of *Asclepias subulata *(cardenolides) have antiproliferative effect on human cancer cells. Cardenolides isolated from *A. subulata *can be used as active chemical markers to elaborate phytopharmaceutical

preparations. The aim of this work was to evaluate the antiproliferative effect of a standardized extract of the aerial parts, based on *Asclepias subulata *cardenolides. **Methods: **Four standardized extracts were prepared by HPLC-DAD depending on the concentration of calotropin and the antiproliferative activity was measured for the MTT assay, on the A549, MCF-7, HeLa, PC3 and ARPE cell lines. The concentrations of calotropin used for the standardization of the extracts were 10, 7.6, 5 and 1 mg/dL. **Results: **Standardization of the *A. subulata *extract based on calotropin at 7.6 mg/g dry weight was achieved and the antiproliferative activity was evaluated over A549, HeLa and MCF-7 cell lines, obtaining proliferation percentages of 3.8 to 13.4% *.* **Conclusions: **The standardized extracts of *A. subulata *at different concentrations of calotropin showed antiproliferative activity against all the cell lines evaluated. The greatest effect was observed against the HeLa cell line.

## Introduction

Cancer continues to be the most aggressive disease and with a high mortality rate. World Health Organization (WHO) estimated that during 2015, 8.2 million people died due to this condition ^1^ but the burden increased in 2018 to 18.1 million new cases and 9.6 million deaths. ^2^ In recent years, there is an increasing interest in the study of natural resources for medicine, so research on the phytochemical, pharmacological and clinical validation of numerous active ingredients derived from natural products has been multiplied. ^3^ Natural products have been considered as a significant source for the generation of pharmaceutical compounds such as analgesics, antifungals, antibiotics, and anticancer agents. ^3^ Herbal medicine has become a non-toxic, safe and readily available source of compounds for cancer treatments ^2^ and the design and research of standardized extracts (preparations obtained only from medicinal herbs that contains pharmacologically active components) can generate advances in the study of new therapies against cancer, reducing or avoiding the appearance of side effects ^3^ since it has been proven that herbal medicine are a safe source, not toxic and readily available cancer treatment compounds. ^2^

*Asclepias subulata* (Asclepiadaceae) is a native plant from the Sonoran Desert in North America, which has been used in traditional medicine for the treatment of several diseases and cancer types. ^4^ In our research group *Rascon et al.,* 2015, ^5^ was demonstrated the antiproliferative activity of the ethanolic extract of the aerial parts of *A. subulata*, with IC50 values of 0.4 and 8.4 ug/mL in A549 and HeLa respectively, and its apoptotic activity was also demonstrated. Additionally, the extract was fractionated to obtain the bioactive compounds, obtaining a new cardenolide 12,16-dihydroxycalotropin and three known compounds, calotropin, corotoxigenin 3-O-glucopyranoside and desglucuzarina. All these compounds showed high antiproliferative activity and selectivity in human cancer cells. ^4^ Despite the most important use of the cardiac glycosides are on cardiac failures treatments, ^6^^,^^7^ recent studies evidenced that cardenolides are also apoptosis inductors and growth-inhibitors against tumors in *in vitro* and *in vivo* models, with no significant toxicity on normal cells. ^6^^,^^8^ These data stimulated us to generate a standardized extract of the aerial parts of *A. subulata* based on its most abundant cardenolides of the plant.

## Methods

### Plants material

*A. subulata* (Decne., 1844) (Asclepiadaceae) was collected in an experimental area located in the Department of Agriculture and Livestock (DAL) of the University of Sonora, Mexico (29°03′18″ North latitude and 111°05′21″ West longitude). The taxonomic identification was carried out by Eng. Jesus Sanchez Escalante and deposited in the Herbarium of the University of Sonora with the voucher number USON-26395.

### Obtaining ethanolic extracts (AsE)

The aerial parts of *A. subulata* were dried at room temperature (25°C), in the shade and grounded with a Whiley mill (200 mesh) affording 300 g of the powder, that was macerated with 70% ethanol in a 1:10 w/v proportion with manual agitation for 10 days. The ethanolic extract was filtered and concentrated on a rotary evaporator at 40 °C under reduced pressure affording 172 g (57.4% yield) of the *A. subulata* etanolic extract (AsE). Chemicals used in supplementary material 2. ^5^^,^^18^

### Design and preparation of standardized extracts

The cardenolides were identified and isolated following the methodology described by Rascon *et al.*, 2015. ^4^ The standardization was performed using a High Performance Liquid Cromatograph (HPLC) (Jasco system compound of binary pump model PU-2086 Plus, São Paulo, SP, Brazil) coupled to a diode array detector (DAD) (Jasco MD-2018 Plus, São Paulo, SP, Brazil) and to an evaporative light scattering detector (ELSD) (Jasco ELS-2040, São Paulo, SP, Brazil). We use a Luna C18 (2) 100A column (250 × 21.2 mm d.i, 5 μm) (Phenomenex, CA, USA) with pre-column (4 × 3 mm d.i.) were used. A binary solvent system was used; solvent A (water with 0.1% formic acid); and solvent B (methanol with 0.1% formic acid) at a flow rate of 7 mL/min ^4^. ^3^^,^^9^ The run was monitored at 30 min. The identification of the chromatographic peaks was performed using addition of compound isolated in the previous step. ESI-IT-MS/MS spectra were obtained with a LTQXL Thermo Scientific spectrometer (ionization mode: negative or positive, scan range: 150–2000 m/z, voltage: –13 kV, heated capillary temperature: 280°C, sheathgas:10 μa, auxiliarygas:10 μa) (San Jose, CA, USA). The NMR experiments were performed on a Bruker Advance 600 MHz equipment, and the samples were processed using CD3OD as the solvent using one and 2D techniques (DEPTQ, HMBC, HSQC, and TOCSY) to confirmation of the structures of the isolated compounds. The calibration curve was prepared using the standard addition method, with seven different concentrations of calotropin (0.3-1 mg/mL) and the values were projected in graph using the PRISMA 5. Considering the IC _50_ of the *in vitro* antiproliferative activity observed in our previous works, ^5^ four standardized extracts were prepared by HPLC-DAD depending on the concentration of calotropin and the antiproliferative activity. The concentrations of calotropin used for the standardization of the extracts were 10, 7.6, 5 and 1 mg/dL, taken by previous studies. ^4^^,^^5^

### Cells lines

A549 (human alveolar adenocarcinoma) (ATCC number: CCL-185), PC-3 (human prostatic adenocarcinoma) (ATCC number: CRL-1435), LS180 (human colorectal adenocarcinoma) (ATCC number: CL-187), HeLa (human cervix adenocarcinoma) (ATCC: CCL-2), ARPE-19 (human retinal pigmented epithelium) (ATCC number: CRL-2302) cell lines were purchased from the American Type Culture Collection (ATCC, Rockville, MD, USA). The cell lines were maintained using Dulbecco’s Modified Eagle’s Medium culture medium supplemented 5% of fetal bovine serum. Cells were grown incubated at 37 °C in a humidified incubator with 5% of CO _2_.

### Antiproliferative activity assay

Antiproliferative activity was evaluated by the MTT reduction assay [3-(4,5-dimethylthiazol-2-yl)-2,5-diphenyltetrazolium] with some modifications, all studies were performed in biological triplicate. ^5^ Briefly, the cells were added into a 96-well plate (1 × 10 ^4^ cells per well in 50 μL of medium). After 24 h incubation at 37°C, were added 50 μL of medium containing different concentrations of standardized extracts: 10 mg/dL; 7.6 mg/dL; 5 mg/dL and 1 mg/dL (maximal DMSO concentration were of 2% v/v) and the cell cultures were incubated for 48 h. Doxorubicin was used as positive control. In the last 4 h of incubation time the cells where were washed with PBS 1X. Fresh culture medium (100 mL) and 10 μL of MTT solution (5 mg/mL) were added to each well. The formed formazan crystals were dissolved with 100 mL of acidic isopropyl alcohol. The absorbance of the samples was measured with an ELISA plate reader (iMark Microplate Absorbance Reader, Bio-Rad), the differences in means were analyzed using one-way analysis of variance (one-way ANOVA) followed by Tukey’s test (Sigma Stat 3; Systat Software Inc., CA, USA).

## Results

### Obtaining ethanolic extracts (AsE)

**Figure 1.**
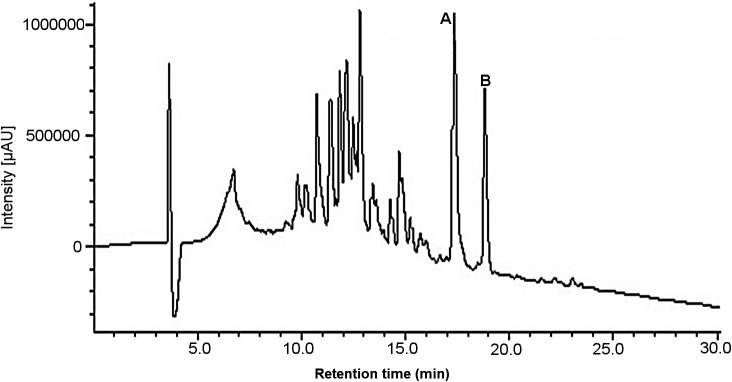
High-Performance Liquid Chromatography chromatogram of *Asclepias subulata* ethanolic extract. A: Calotropin (17.4 min), B: Calactin (18.1 min).

The AsE was prepared by maceration for 10 days with 70% ethanol, filtered and dried, were yielding of 172.1 g (57.4%). Fractionation of 5 g of AsE using gel permeation chromatography on an open column of Sephadex and further purification using a semipreparative HPLC-DAD system led to the isolation of the cardenolides ( Figure 1), which were identified using NMR, Mass spectrometry and comparison to previous literature data. ^4^ Total of 9 subfractions were obtained and then were analized for subjected thin layer chromatography (TLC) technique and HPLC to know the complexity of its compounds.

Thus, fractions 3 (1167.2 mg) and 4 (576.6 mg) were selected and gave us two signals called compound A and compound B, which shown a maximum absorbance between 210 and 225 nm and it is reported that a maximum absorption between 214 and 222 nm correspondence to a functional group of unsaturated lactones, considered cardenolides; ^10^ the structures were confirmed by NMR and agree with the data reported by the bibliography. ^4^ The chromatographic profiles were optimized by means of analytical HPLC and subsequently the conditions used were extrapolated to a semi-preparative HPLC system using the ELSD as the main detector due to the low absorption in the UV-visible spectrum that the samples presented. This way it was possible to obtain compounds 1, 2 and 3. Compound 1 (Rt 17.4 min **)** was obtained as a fine white powder (32.8 mg). Its ESI-MS mass spectrum showed an [M-H] ^-^ ion at m/z 531; comparison with the literature allowed us to identify 1 as calotropin. ^4^ Compound 2 (Rt 18.1 min) generated amorphous and colorless crystals with a weight of 21.2 mg, Its ESI-MS mass spectrum showed an [M-H] ^-^ ion at m/z 577; comparison with the literature allowed us to identify 2 as calactin. ^11^ Compound **3 (**R _T_ 17.9 min) was obtained as a fine white powder (5 mg). Its ESI-MS mass spectrum showed the [M-H] ^-^ ion at m/z 547. MS3 fragmentarion of the precursos ion at m/z 547 led to the product iosn at m/z 529, 511 and 401, which are in accordance with Rascon *et al.*, 2015. ^4^ This compound corresponds to cardenolide 4′-hydroxy-7,8-dehydrocalotropin. All these compounds were also subjected to NMR analyses and compared to literature data ^4^ to confirm their identities (see extended data for more information ^17^). Continuing with the study, by means of mass spectrophotometry the identification of 11 compounds shown in Supplementary material 2 (see extended data ^17^) was achieved.

### Design and preparation of AsE

**Figure 2.**
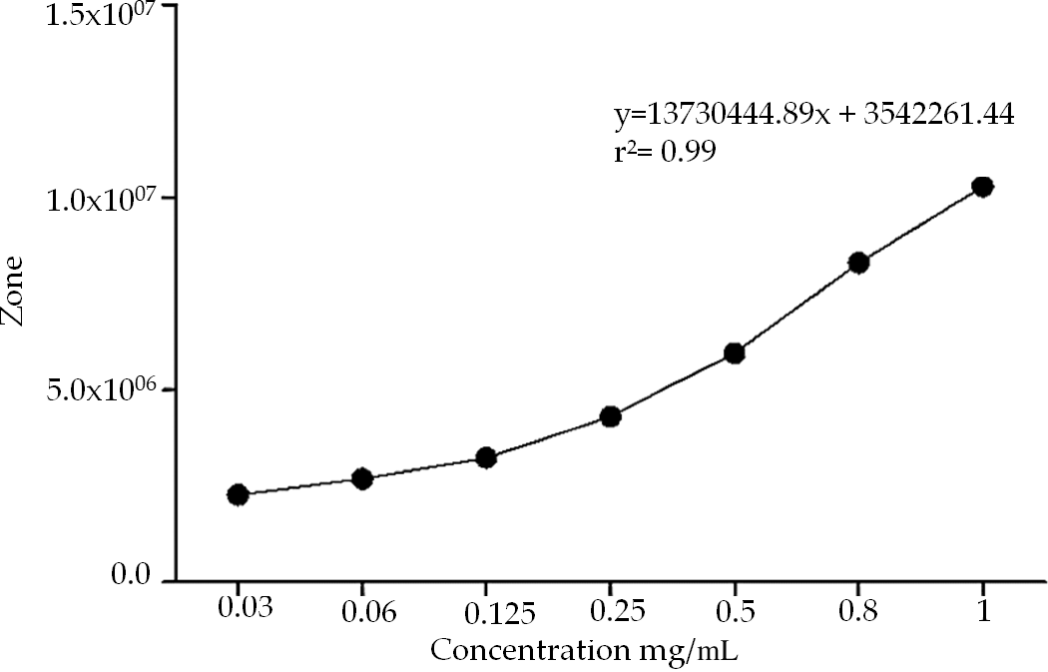
Calibration curve of phytopreparation from *Asclepias subulata.* Calotropin was quantified using different concentrations (0.3-1 mg/mL) whit standard addition method by HPLC.

Plants in a natural environment face different biological and ecological stimuli that mean that they do not provide secondary metabolites in a consistent way; since nature does not provide a product with consistent or standardized concentrations, it is necessary to perform a standardization of extracts that are required to be used in traditional medicine, but the standardization of an herbal product is complicated, starting from its cultivation, extraction, storage, etc. ^12^ After the clean-up, the AsE was analyzed using HPLC-DAD-ELSD ( Figure 1). The chromatographic run was achieved in about 30 min and led to a good resolution of the peaks assigned to compounds 1 and 2, which is essential for the standardization of the AsE. Standardization of the AsE led to the final concentration of 7.6 mg of calotropin per gram of AsE ( Figure 2). Once this information was gathered, we were able to use three different concentrations of the extract based on calotropin and IC _50_ and the IC _50_ reports present in the previous studies: a low (1 mg/dL), a medium (5 mg/dL), a high (10 mg/dL) and a base 7.6 mg/dL that allowed us to continue with the evaluation of the extract in subsequent experiments.

Antiproliferative activity of the AsE

Then, based on this result and the IC _50_ values reports reported in our previous studies we were able to investigate the antiproliferative activity of AsE using four different concentrations: 10 mg/dL (high); 7.6 mg/dl (base); 5 mg/dL (medium) and 1 mg/dL (low), which were evaluated in different cell lines ( Table 1), HeLa being the most affected; a maximum of 10% proliferation was observed in all lines, which shows that the extract even in the low concentration of 1 mg/dL stops the growth of cancer cells *in vitro.*

**Table 1.** Percentage of proliferative activity of the phytopreparations of *Asclepias subulata.*

Concentration of calotropin in phytopreparations		Cell Lines (% Proliferation)		
	ARPE-19	A549	HeLa	MCF-7
10 mg/dL	37.0 ± 1.6 ^a^ ^d^	7.9 ± 0.7 ^b^ ^d^	4.4 ± 0.6 ^b^ ^d^	10.2 ± 0.7 ^b^ ^d^
7.6 mg/dL	38.1 ± 1.1 ^a^ ^d^	7.9 ± 0.6 ^b^ ^d^	5.1 ± 0.9 ^b^ ^d^	10.6 ± 0.8 ^b^ ^d^
5 mg/dL	42.6 ± 2.1 ^a^ ^d^	9.0 ± 0.9 ^b^ ^d^	5.3 ± 1.0 ^b^ ^d^	11.1 ± 1.0 ^b^ ^d^
1 mg/dL	47.2 ± 0.9 ^a^ ^d^	10.0 ± 0.7 ^b^ ^d^	6.0 ± 0.9 ^b^ ^d^	11.4 ± 1.0 ^b^ ^d^

A549: Non-small cell lung cancer (NSCLC). MCF-7: Breast adenocarcinoma. HeLa: Adenocarcinoma of the cervix. ARPE-19: retinal pigment epithelium.

^a^ Statistically significant difference at *p*=0.05. ± Standard deviation.

^b^ There is no statistically significant difference *p*=0.05. Relationship of antiproliferative activity of cancer cells with respect to the non-cancerous cell line.

^c^ Values <1; Selectivity on the normal cell line.

^d^ Values >1; Selectivity on cancer cell line. The values obtained represent three independent experiments ± standard deviation.

## Discussion

The term cancer includes more than 100 different types of diseases that feature the accelerated and disorderly growth of cells with abnormal expressed genes that participate in the regulation of the cell cycle directly, ^13^ with an incidence of 439.2 cases per 100 thousand inhabitants of which 163.5 will die from the disease. ^1^ The generation of standardized extracts that support the treatments against this disease and given its high mortality and worldwide incidence is of vital importance. In the present work, it was standardized by HPLC-DAD technique, using calotropin as internal standard; the AsE at a concentration of 7.6 mg/g of dried plant, presented an antiproliferative activity with less than 1 ug/mL showing cytotoxic effect in different cell lines, being the most sensitive HeLa and A549, Table 2. The selectivity of the extract for cancer lines is evident when comparing the activity in Table 2, since the ARPE-19 line (non-cancerous line used as control) has a lower sensitivity than cancer lines, this can be explained due to the state of massive and uncontrolled proliferation of the cancer cells and its requirements for different signaling pathways that favor their high proliferation metabolism, conveniently changing their energy production from one pathway to another, such as the overexpression of Na ^+^/K ^+^ pumps; epidermal growth receptors (EGFR), insulin receptors, etc. ^14^ Therefore, it is hypothesized that the compounds present in the *A. subulata* extract could be inhibit some growth receptors over-expressed in these cell lines, such as EGFR. Rascon *et al.*, 2015, reported the antiproliferative activity of the methanol extract with IC _50_ values in A549 (8.7 μg/mL) and HeLa (<0.4 μg/mL) cell lines; ^5^ in the present study, an increase in activity was achieved following the activity patterns reported by Rascon *et al*. 2015 reporting the increase in antiproliferative activity when using an ethanolic fraction, ^5^ which was verified by generating an ethanolic extract and observing the increase in antiproliferative activity at 0.23 μg/mL in A549 and 0.6 in HeLa, respectively, Table 1. This is due to the concentration of secondary metabolites (cardenolides) in the fraction. The antiproliferative activity of *A. subulata* extracts can differ if its wild or from an artificial culture, and this is demonstrated comparing the IC _50_ values reported by Bustamante *et al.,* 2020 (IC _50_ 0.8 μg/mL), ^15^ Rascon *et al.,* 2015 (IC _50_ 8.7 μg/mL) ^5^ and the present work (IC _50_ 0.23 μg/mL). This can be explained because in conditions of natural stress the wild plant does not modify the production of natural metabolites, but in crops where these stress levels are modified, such as harvest season, water stress and others, the best growing conditions can increase the levels of the secondary metabolites and thus generate an extract with greater antiproliferative activity. Bustamante *et al.,* 2020 reported a calotropin production (reference cardenolide) of 236. 97 μg/g average of the generated crop, while in this study we reported a calotropin concentration of 7.6 mg/g of dried plant. The difference between calotropin concentration can be influenced by different biotic stress factors such as depredation of the plant by worms of the monarch butterfly and the false monarch at the harvest time of the plant; Agrawal *et al.* 2014 reported that the plant increases the production of cardenolides as a defense mechanism, demonstrated by the increase in the cardenolides of *Asclepias sicaria* and *Asclepias halli* when they were stimulated by depredation of monarch butterfly worms. ^16^

**Table 2.** Antiproliferative activity of the ethanolic extract of *Asclepias subulata.*

	ARPE-19	Cell Lines (IC _50_ μg/mL)		
		A549	HeLa	MCF-7
Ethanolic extract of *Asclepias subulata*	>20.0	0.23 ± 0.03	0.6 ± 0.2	0.5 ± 0.03
Doxorubicin *	1.02 ± 0.12	1.7 ± 0.12	>4.0	0.7 ± 0.04

A549. Non-small cell lung cancer (NSCLC). MCF-7. Breast adenocarcinoma. HeLa. Adenocarcinoma of the cervix. ARPE-19: retinal pigment epithelium.

* Positive control.

The method of quantification of cardenolides is another factor to consider, for his part Bustamante *et al.,* 2020 uses an external standardization pattern while in the present work an internal one was used, managing to eradicate the error of the matrix. It has been shown that the antiproliferative activity of the extracts from *Asclepias subulata* given by its bioactive principles the cardenolides such as: calotropin, calactin; 4′-hydroxy-7,8-dehydrocalotropin; identified in the plant (supplementary material 2) ^4^ that can interact with the Na ^+^/K ^+^ pump and cell death receptors, caspase activation and mitochondrial membrane depolarization, among other signaling pathways for the generation of cellular apoptosis. ^8^^,^^17^ Studies by Rascon *et al.* 2015 demonstrated that the *Asclepias subulata* extract could induce cell apoptosis through the activation of caspases 3, 8 and 9 and the depolarization of mitochondria, hypothesizing that the activation of apoptosis occurs intrinsically. ^4^^,^^5^^,^^8^ More recent studies have shown that cardenolides can activate various metabolic pathways that induce apoptosis, the most defined mechanism being the interaction they have with the Na ^+^/K ^+^ pump, since when interacting with it they generate a conformational change that induces the activation of the protein. c-Src tyrosine kinase, which interacts with the epidermal growth receptor (EGFR) and the activation of the Ras-Raf pathway by activating the protein by phosphorylation Ras which in turn initiates the downstream signaling pathway of Raf, Mek and Erk that leads to the production of reactive oxygen species and activation of pro-apoptotic proteins such as Bax and the decline of anti-apoptotic proteins of the BCL-2 family, the depolarization of the mitochondrial membrane and activation of caspases, activation of cycle arrest proteins in cells such as p53 and p21 that together act for the activation of cellular apoptosis. ^4^^,^^5^^,^^8^^–^^10^ It is hypothesized that this pathway is the mechanism of action of the generated extract and to achieve its clarification it is of vital importance to evaluate the proteins involved such as p53, Bax, Ras, Raf among others to establish it.

## Conclusions

An ethanolic extract of *Asclepias subulata* was generated with a concentration of 7.6 mg of calotropin per gram of dry weight with IC _50_ less than 1 μg/mL in different cell lines, presenting better activity in cell lines such as HeLa and A549, which places it as an extract with activity antiproliferative candidate for generation of phytopreparation against cancer in the near future, it is recommended to evaluate the route of action both *in vitro* and *in vivo* to reinforce the acceptance and generation of the phytopreparation.

## Data availability

### Underlying data

Open Science Framework: Antiproliferative activity of standardized herbal phytopreparation from *Asclepias subulate*, https://doi.org/10.17605/OSF.IO/NC5V3. ^18^

This project contains the following underlying data:

- Mass data.rar
- Francisco_4′-hydroxy-7,8-dehydrocalotropin.zip
- Francisco_Calactin.zip
- Francisco_Calotropin.zip
- Calibration curve data

### Extended data

Open Science Framework: Antiproliferative activity of standardized herbal phytopreparation from *Asclepias subulate*, https://doi.org/10.17605/OSF.IO/NC5V3. ^18^

This project contains the following extended data:

- Compounds identified by HPLC-MS from ethanolic extract of Asclepias subulata.docx
- Spectrometric data from 1H-RMN y 13C-RMN for compounds A and B in CD3OH δ expressed in ppm.docx

Data are available under the terms of the Creative Commons Attribution 4.0 International license (CC-BY 4.0).
