# Supplementary material for: Antiproliferative activity of standardized herbal phytopreparation from Asclepias subulata
Source: F1000Res. 2022 Oct 25;11:527. Originally published 2022 May 16. [Version 2] doi: 10.12688/f1000research.111181.2 (PMC10071139; doi:10.12688/f1000research.111181.2)
Supplement: Peer review history

# Peer review: Reviewer response for version 2

**DOI:** 10.5256/f1000research.139179.r166233

**Published:** 2023-04-03

**Authors:** Ángel Trigos

**Affiliations:** 1 Centro de Investigación en Micología Aplicada, Universidad Veracruzana, Xalapa, Veracruz, 91010, Mexico

The manuscript entitled: "Antiproliferative activity of standardized herbal phytopreparation from *Asclepias subulata*" is a well-structured paper and reports interesting results on the standardization of a  phytopharmaceutical preparation based on *Asclepias subulata* with antiproliferative activity on some cancer cell lines.

 However, from my point of view, I suggest some observations that could enrich the manuscript.

 a) In order not to confuse the reader, I request that the idea that medicinal plants are not toxic be corrected.

 b) Although the reported results are promising, since the standardized extracts of *Asclepias subulata* presented anti-proliferative activity against some cancer cell lines, I wonder if these extracts could have side effects related to heart function? I suggest discussing it briefly.

 c) In the discussion, it refers to the fact that cancer is a group of diseases and after that, it is a disease. Please correct.

 It is important, and also with the aim of not confusing the reader, that it be clarified that in order to use this phytopharmaceutical preparation in the future, specific antitumor evaluation tests would be lacking, in addition to the toxicity tests, check the side effects related to the functioning of the heart, studies pharmacokinetics and pharmacodynamics, etc.

 Therefore, once the authors make these brief suggestions, I consider this MS can be indexed.

Is the work clearly and accurately presented and does it cite the current literature?

Yes

If applicable, is the statistical analysis and its interpretation appropriate?

Yes

Are all the source data underlying the results available to ensure full reproducibility?

Yes

Is the study design appropriate and is the work technically sound?

Yes

Are the conclusions drawn adequately supported by the results?

Partly

Are sufficient details of methods and analysis provided to allow replication by others?

Yes

Reviewer Expertise:

Chemistry of natural products (mushrooms)

I confirm that I have read this submission and believe that I have an appropriate level of expertise to confirm that it is of an acceptable scientific standard.

# Peer review: Reviewer response for version 2

**DOI:** 10.5256/f1000research.139179.r154145

**Published:** 2022-10-26

**Authors:** Juan Carlos Sepúlveda-Arias

**Affiliations:** 1 Programa de Doctorado en Ciencias Biomédicas, Facultad de Ciencias de la Salud, Universidad Tecnológica de Pereira, Pereira, Colombia

The authors improved the manuscript according to my suggestions.

Is the work clearly and accurately presented and does it cite the current literature?

Partly

If applicable, is the statistical analysis and its interpretation appropriate?

Partly

Are all the source data underlying the results available to ensure full reproducibility?

Yes

Is the study design appropriate and is the work technically sound?

Yes

Are the conclusions drawn adequately supported by the results?

Yes

Are sufficient details of methods and analysis provided to allow replication by others?

Yes

Reviewer Expertise:

Biological activity of natural products, Immunomodulation, Immunology, Molecular Biology, Biotechnology.

I confirm that I have read this submission and believe that I have an appropriate level of expertise to confirm that it is of an acceptable scientific standard.

# Peer review: Reviewer response for version 1

**DOI:** 10.5256/f1000research.122874.r139841

**Published:** 2022-06-21

**Authors:** Juan Carlos Sepúlveda-Arias

**Affiliations:** 1 Programa de Doctorado en Ciencias Biomédicas, Facultad de Ciencias de la Salud, Universidad Tecnológica de Pereira, Pereira, Colombia

González Gutierrez *et al*. did evaluate the antiproliferative activity of a standardized ethanolic extract obtained from the aerial parts of *Asclepias subulata*. The extract was standardized base on different concentrations of calotropin. The work presented is part of a research line aimed at studying the composition and biological activity of extract obtained from *Asclepias subulata* from the región of Sonora, as evidenced by previous publications. I consider that the manuscript is important and interesting. However, I have some minor considerations:

1. The concentrations of calotropin in the extracts used for the standardization are presented in the abstract as 'mg/dL', in methods as 'mg/mL', and in the antiproliferative activity section also as 'mg/dL' - please correct.
2. I suggest including in table 1 the Selectivity Index values.
3. Please include the number of experiments and replicates used to evaluate the antiproliferative activity. Also, I suggest revising the statistical analysis and considering using the Kruskal-Wallis test with Dunn´s post hoc test.
4. Please edit the language and grammar because some parts are difficult to follow (please revise the annotated copy of the article I have attached here).

Is the work clearly and accurately presented and does it cite the current literature?

Partly

If applicable, is the statistical analysis and its interpretation appropriate?

Partly

Are all the source data underlying the results available to ensure full reproducibility?

Yes

Is the study design appropriate and is the work technically sound?

Yes

Are the conclusions drawn adequately supported by the results?

Yes

Are sufficient details of methods and analysis provided to allow replication by others?

Yes

Reviewer Expertise:

Biological activity of natural products, Immunomodulation, Immunology, Molecular Biology, Biotechnology.

I confirm that I have read this submission and believe that I have an appropriate level of expertise to confirm that it is of an acceptable scientific standard.

# Response

**Published:** 2022-09-27

**Authors:** Ramón Enrique Robles Zepeda

**Affiliations:** Universidad de Sonora, Mexico

Firstly, we would like to thank you for considering our work for indexing and secondly, thank you for your comments, which we allow ourselves to refute in some cases and follow up and change for your advice. Next, we present each of them:

**1. The concentrations of calotropin in the extracts used for the standardization are resented in the abstract as 'mg/dL', in methods as 'mg/mL', and in the antiproliferative activity section also as 'mg/dL' - please correct.**

 Thanks for the comments, regarding the different measurement units used, they are correct, to generate a phytopreparation with visualization to be used in in vivo models, it was necessary to use units of mg/dL, however in the in vitro evaluation and for the standardization of the phytopreparation it is necessary to use units in mg/mL.

**2. I suggest including in table 1 the Selectivity Index values.**

 It was included.

**3. Please include the number of experiments and replicates used to evaluate the antiproliferative activity. Also, I suggest revising the statistical analysis and considering using the Kruskal-Wallis test with Dunn´s post hoc test.**

 Thank you for noting the lack of information, this has already been attached (highlighted in red); Based on the statistical analysis, the analysis of variance test was carried out using the Tukey test, due to the type of study and the type of data generated.

**4. Please edit the language and grammar because some parts are difficult to follow (please revise the annotated copy of the article I have attached here).**

 Thank you for providing us with the language fixes, they were all taken into consideration and modified.

# Peer review: Reviewer response for version 1

**DOI:** 10.5256/f1000research.122874.r137993

**Published:** 2022-06-07

**Authors:** Shadma Wahab

**Affiliations:** 1 Department of Pharmacognosy, College of Pharmacy, King Khalid University, Abha, Saudi Arabia

Activity against cell proliferation of a standardized herbal Phyto preparation derived from Asclepias subulata has been reported in this study. After reviewing, I found the noticeable strengths in the article; however, the study needs Major revision before final acceptance.

 Further, my comments on the article are as follows:

1. What is the rationale for selection for the proposed antiproliferative activity? The introduction section can be revised to justify the type of activity preferred and the rationale behind that.
2. Why has the author chosen aerial parts of *A. subulata *for *a*ntiproliferative activity?
3. Why was the maceration method selected to prepare the extract? Please put the appropriate reference for the chosen method.
4. Information on the chemicals used in the study is missing.
5. Why was the solvent ethanol selected as extracting solvent? Why not any other solvent? 
6. Discussion should be the interpretation of the results, not just the explanation of the results. Hence must be result-oriented.
7. Please revise the conclusion section, and future recommendations should be included.
8. Add some recent references in the methodology part.

Is the work clearly and accurately presented and does it cite the current literature?

Partly

If applicable, is the statistical analysis and its interpretation appropriate?

Partly

Are all the source data underlying the results available to ensure full reproducibility?

Partly

Is the study design appropriate and is the work technically sound?

No

Are the conclusions drawn adequately supported by the results?

No

Are sufficient details of methods and analysis provided to allow replication by others?

Yes

Reviewer Expertise:

Isolation and Identification of natural products, Molecular Docking, anticancer, antifungal, antibacterial antidiabetic activities of plants and their constituents.

I confirm that I have read this submission and believe that I have an appropriate level of expertise to state that I do not consider it to be of an acceptable scientific standard, for reasons outlined above.

# Response

**Published:** 2022-09-27

**Authors:** Ramón Enrique Robles Zepeda

**Affiliations:** Universidad de Sonora, Mexico

Firstly, we would like to thank you for considering our work for indexing and secondly, thank you for your comments, which we allow ourselves to refute in some cases and follow up and change for your advice. Next, we present each of them:

**1.- What is the rationale for selection for the proposed antiproliferative activity? The introduction section can be revised to justify the type of activity preferred and the rationale behind that.**

 Thank you for considering the reading of the article and its corrections; Considering point number one, we allow ourselves to differ from your comment since the introduction is handled based on the collaborative work of our working group where the high antiproliferative activity of the phytopreparation is specified, which is reflected in articles cited in the publication, such as: Rascon *et al *2015.

**2.- Why has the author chosen aerial parts of *A. subulata* for antiproliferative activity?**

 In consideration of this question, in our work we are trying to establish the potential of a phytopreparation of an endemic plant from the northwest of the state of Sonora; whereby; the total deforestation of the plant is not convenient for our future work; Likewise, it is known that the main secondary metabolites of the plant (cardenolides) are distributed in the aerial part of the plant, since they are used as a defense mechanism against predation.

 Agrawal A. Hastings A. Patrick E. Knight A. Specificity of Herbivore-Induced Hormonal Signaling and Defensive Traits in Five Closely Related Milkweeds ( *Asclepias spp.)* J ChemEcol. 2014.1-13. Doi: 10.1007/s10886-014-0449-6.

**3.- Why was the maceration method selected to prepare the extract? Please put the appropriate reference for the chosen method.**

 I appreciate the correction of the bibliographic citation, which was added to the text, as well as the greater detail of the technique used.

**4.- Information on the chemicals used in the study is missing.**

 In consideration of this point, the information on each of the chemicals used in the study was added to the complementary material part, thanks for noting the lack of information.

**5.- Why was the solvent ethanol selected as extracting solvent? Why not any other solvent?**

 In our group, we worked with methanolic extracts of *Asclepias subulata* (Rascon et al. 2015), as well as its fractions with different solvents changing their polarity; It was observed that the one with the best biological activity was the ethanolic fraction, as well as in which the bioactive compounds were found, for this reason we worked with this fraction. Later, Bustamante *et al*., 2020, established the conditions to be able to work with the ethanolic extract and the concentration of the bioactive compounds. Based on these two works, the decision was made to work with the same ethanolic extract that are expressed in the work under review.

**6.- Discussion should be the interpretation of the results, not just the explanation of the results. Hence must be result oriented.**

 In reference to this point, the discussion was improved both in terms of writing and information. Thanks for your opinion.

**7.- Please revise the conclusion section, and future recommendations should be included.**

 Considering this point, the conclusion was modified giving a final point of recommendation following the mentioned point of view.

 An ethanolic extract of *Asclepias subulata* was generated with a concentration of 7.6 mg of calotropin per gram of dry weight with IC50 less than 1 μg/mL in different cell lines, presenting better activity in cell lines such as HeLa and A549, which places it as an extract with antiproliferative activity that is a candidate for the generation of a phytopreparation against cancer in the near future, it is recommended to evaluate the route of action both *in vitro* and *in vivo* to reinforce the acceptance and generation of the phytopreparation.

**8. Add some recent references in the methodology part.**

 1.- Vidal M, Torres H, Hernandez S, et al.: Antiproliferative activity of standardized phytopreparations from Ibervillea sonorae (S. Watson) Greene. Steroids. 2021; 169: 108824.

 2.- Wambua J, Wafula P, Muhoro J, Naulikha S.: In vitro anti-cancer efficacy and phyto-chemical screening of solvent extracts of Kigelia africana (Lam.) Benth. 2020; e04481. https://doi.org/10.1016/j.heliyon.2020.e04481
